# Genomic Composition of the Artificial Hybrid ×*Trititrigia cziczinii* (Hordeeae, Poaceae) and Related Taxa According to Molecular Phylogenetic Data

**DOI:** 10.3390/plants15010070

**Published:** 2025-12-25

**Authors:** Alexander A. Gnutikov, Nikolai N. Nosov, Evgeny V. Zuev, Natalia S. Lysenko, Victoria S. Shneyer, Aleksey V. Troitsky, Alexander V. Rodionov

**Affiliations:** 1 N.I. Vavilov Institute of Plant Genetic Resources (VIR), St-Petersburg 190000, Russia; a.gnutikov@vir.nw.ru (A.A.G.); e.zuev@vir.nw.ru (E.V.Z.); n.lysenko@vir.nw.ru (N.S.L.); 2 Komarov Botanical Institute (BIN RAS), St-Petersburg 197022, Russia; shneyer@binran.ru (V.S.S.); avrodionov@binran.ru (A.V.R.); 3 Belozersky Institute of Physico-Chemical Biology, Lomonosov Moscow State University, Moscow 119992, Russia; bobr@belozersky.msu.ru

**Keywords:** genome elimination, *Elytrigia intermedia*, intergeneric hybridization, next-generation sequencing (NGS), rDNA, ribotype, *Triticum aestivum*

## Abstract

×*Trititrigia cziczinii* Tzvelev is a promising crop developed through distant hybridization between *Elytrigia intermedia* (Host) Nevski (=*Thinopyrum intermedium* (Host) Barkworth & D.R. Dewey) and *Triticum aestivum* L., followed by backcrossing with wheat. This study elucidates the genomic composition of this hybrid and its parental taxa using molecular phylogenetic analysis of nuclear (ITS, ETS) and chloroplast (*trn*K–*rps*16, *ndh*F) DNA markers, complemented by Next-Generation Sequencing (NGS) of the 18S–ITS1–5.8S rDNA region. Results from Sanger sequencing revealed that the primary nuclear ribosomal DNA (rDNA) of the hybrid originates from *Triticum aestivum*; a finding strongly supported by both Bayesian inference and Maximum Likelihood analyses. Chloroplast DNA data unequivocally indicate maternal inheritance from *T. aestivum*. In contrast, ETS sequence analysis showed phylogenetic affinity to *Elytrigia intermedia*, suggesting complex genomic reorganization or chimeric sequence formation in the hybrid. NGS data corroborate the dominance of *T. aestivum*-like ribotypes in the hybrid’s rDNA pool, with only a minor fraction identical to the main ribotype of *E. intermedia*. Genetic structure analysis further revealed geographic heterogeneity in the genomic composition of *E. intermedia* populations. The predominance of the wheat genome in ×*T. cziczinii* is likely a consequence of stabilizing backcrosses and illustrates a case of rDNA elimination from one parental genome during hybridization. This research underscores the complex genomic dynamics in artificial hybrids and the utility of multi-marker phylogenetic approaches for clarifying their origins.

## 1. Introduction

As is well known, interspecific and intergeneric hybridization is widespread in angiosperms. For a long time, evidence of a species’ hybrid origin was considered to be the presence of morphological traits shared by other species growing nearby, or an intermediate state of some morphological traits between the putative parents. After it became possible to study molecular traits and compare genomic regions, it was discovered that in some plants, the DNA sequence of the compared region contains traits (nucleotides) from two different species, indicating that the plant is a hybrid. However, sometimes the presence or intermediate state of the parental morphological traits is weakly expressed or completely absent.

The study of hybrids at the genetic level is of great importance. The genetic pattern of hybrids can be complex, as it can be represented by two parental subgenomes, but sometimes the subgenome of one parent can be eliminated. Furthermore, a hybrid can undergo backcrosses with one (or both) parents, and such introgression processes influence the genomic constitution.

A better understanding of the structure of hybrid genomes can be achieved by studying artificial hybrids obtained specifically. Distant hybridization has very broad potential for harnessing the genomic potential of wild relatives to improve existing crop varieties and develop new ones for agricultural production. There is currently a tendency to crossbreed in a narrow range of varieties used as source material in wheat breeding. Continuing this trend leads to genetic erosion: loss of genetic diversity, including genes and entire gene complexes that are characteristic of plants adapted to certain environmental conditions [1,2,3]. This leads to the impoverishment of the genetic base of the agricultural crop varieties used and developed [1]. N. I. Vavilov recommended involving both geographically distant wheat accessions and wild-growing relatives in selection to expand the wheat gene pool [4].

For our molecular phylogenetic analysis, we took ×*Trititrigia cziczinii* Tzvelev (tribe Hordeeae Martinov = Triticeae Dumort.), cultivar “In Memory of Lyubimova”. It is known that this variety was created as a result of intergeneric hybridization of couch grass *Elytrigia intermedia* (Host) Nevski (=*Thinopyrum intermedium* (Host) Barkworth & D.R. Dewey) and *Triticum aestivum* L. with subsequent backcrosses with *Triticum aestivum*. According to morphological features, it occupies an intermediate position between the parent taxa with a bias towards wheat features [5,6,7]. Its genome contains 56 chromosomes (42 from wheat and 14 from couch grass) [6]. A distinctive feature of this variety is the intensive growth of new shoots after ripening and harvesting of a grain, which allows, under favorable conditions, for obtaining both a grain harvest and a green mass harvest during the growing season [6]. In addition, it has a high content of protein and gluten in the grains [5]. It is resistant to fusarium, smut, and brown rust [6]. All these features allow us to consider this hybrid a promising agricultural crop, and it is important to know in detail its genome composition.

We used for an analysis the nuclear ITS1–5.8S rDNA–ITS2, 18S rDNA–ITS1–5.8S rDNA, and 3′-ETS regions as well as chloroplast *trn*K–*rps*16 and *ndh*F sequences. The level of their variation is suitable for evolutionary studies at the species level; ITS and *trn*K–*rps*16 spacers are often applied for DNA barcoding [8,9]. ITS sequences, despite their susceptibility to homogenization, have often been used for phylogenetic reconstruction in complicated and highly hybridized groups for almost a quarter of a century [10,11,12,13]. External transcribed spacer (ETS) of rDNA is closely related to ITS, evolves at a similar rate and is regarded as a useful tool for phylogenetic analysis though is used less frequently [14]. Chloroplast sequences are mostly inherited uniparentally through the maternal line [15,16]. Thus, these sequences can be used for phylogenetic reconstruction of the barley tribe species (Hordeeae) where hybridization is widely distributed and is one of the main evolutionary pathways [17,18,19,20]. Chloroplast sequences *trn*K–*rps*16 and *ndh*F usually evolve more slowly than ITS, but they are invaluable for constructing independent phylogenies in the case of introgressive hybridization. In addition, NGS technologies now make it possible to obtain the entire pool of the marker sequences, including hidden ones left over from ancient hybridization processes and not detectable by other methods, including cloning. For example, intragenomic polymorphism of different sequences was studied in genera *Hordeum* L., *Nepenthes* L., and *Abies* Mill. [21,22,23].

Given the above, our aim was to establish the possible genomic combination of the hybrid ×*Trititrigia cziczinii* and compare it with those of their parental taxa using marker sequences of the nuclear and chloroplast genomes.

## 2. Results

### 2.1. Sanger Sequencing Data

**Figure 1:**
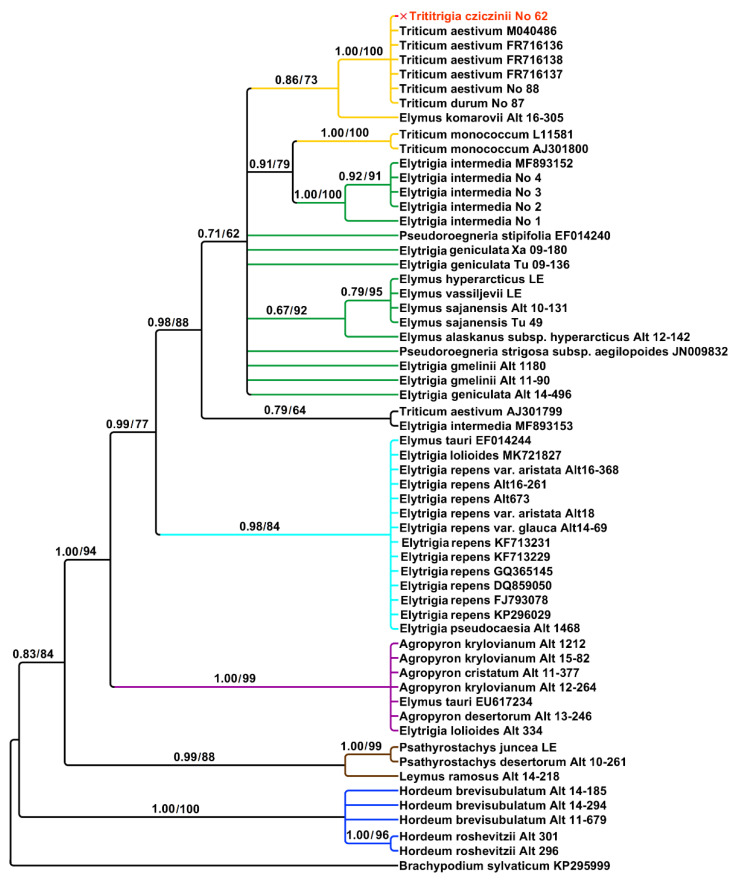
Phylogenetic tree showing the relationships of artificial hybrid ×*Trititrigia cziczinii*, parental taxa, and allied genera according to the ITS data (Sanger method). The first index near the tree nodes is the posterior probability in Bayesian inference, the second is bootstrap value calculated by Maximum Likelihood analysis.

A list of the studied species is given in Table 1. The region ITS1–5.8S rDNA–ITS2 contains 613 aligned positions. Analysis of ITS sequences that were obtained by Sanger method clearly indicated the origin of the main rDNA in ×*Trititrigia cziczinii* from *Triticum aestivum*. *Triticum durum* Desf. also fell within this subclade (PP = 1, BS = 100) (Figure 1). The subclade of ×*Trititrigia cziczinii*, *Triticum aestivum*, and *T. durum* is sister to *Elymus komarovii* (Nevski) Tzvelev in moderately supported clade (PP = 0.86, BS = 73).

The tree is moderately to strongly supported (Figure 1). According to the ITS data, not only different genera but also rDNA sequences of the different genomes formed separate clades. Diploid *Triticum monococcum* L. fell outside the ×*Trititrigia* Tzvelev + *T. aestivum* clade and was monophyletic with samples of *Elytrigia intermedia* (PP = 0.91, BS = 79). Studied samples of *Triticum* and ×*Trititrigia cziczinii* form one large clade with Arctic species of *Elymus, Elytrigia gmelinii*, and *Pseudoroegneria stipifolia* (PP = 0.71, BS = 62). Arctic species of *Elymus* form a weakly to moderately supported subclade (PP = 0.67, BS = 92). *Triticum aestivum* and *Elytrigia intermedia* from Genbank form a separate clade (PP = 0.79, BS = 64). *Elytrigia repens* (L.) Nevski (=*Elymus repens* (L.) Gould) forms a strongly supported clade with *Elytrigia lolioides* (Kar. & Kir.) Nevski, *E. pseudocaesia* (Pacz.) Prokudin, and *Elymus tauri* (Boiss. & Balansa) Melderis (PP = 0.98, BS = 84). Studied samples of *Agropyron krylovianum* Schischk., *A. cristatum* (L.) Gaertn., and *A. desertorum* (Fisch. ex Link) Schult. are placed in a maximally supported clade with the samples of *Elytrigia lolioides* and *Elymus tauri* (PP = 1, BS = 0.99) probably forming a clade of P-genome. Samples of *Psathyrostachys* Nevski are monophyletic with *Leymus* Hochst. (PP = 0.99, BS = 88), studied samples of the genus *Hordeum* L. (subgenus *Critesion* (Raf.) Tzvelev) are also monophyletic (PP = 1, BS = 100).

**Figure 2:**
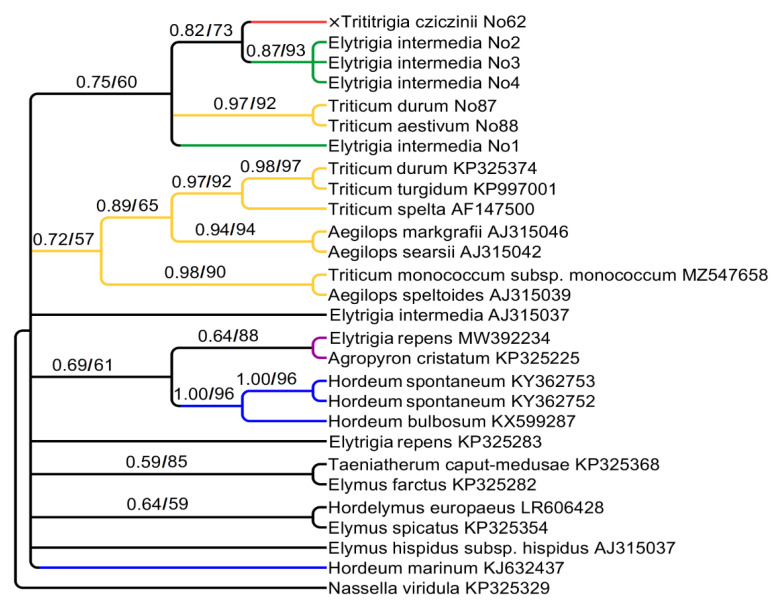
Phylogenetic tree showing the relationships of artificial hybrid ×*Trititrigia cziczinii,* parental taxa, and allied genera according to the ETS data (Sanger method). The first index is the posterior probability in Bayesian inference, the second is bootstrap value calculated by Maximum Likelihood analysis.

We analyzed 3′-ETS of 18S rDNA. Its alignment consists of 303 positions. The ETS tree (Figure 2) is less supported than the previous one. Unexpectedly, ETS sequences of ×*Trititrigia cziczinii* turned to be sister to *Elytrigia intermedia* (PP = 0.82, BS = 73). ETS sequence of *Triticum aestivum* groups with *T. durum* (PP = 0.97, BS = 92) and does not group with ×*Trititrigia cziczinii.* Other *Triticum* samples from Genbank database fall into a clade with *Aegilops* L. according to ETS data. Additionally, *Elytrigia repens* groups with *Agropyron cristatum* (probably P-genome). ETS sequences of *Hordeum* species enter three different clades on the tree.

**Figure 3:**
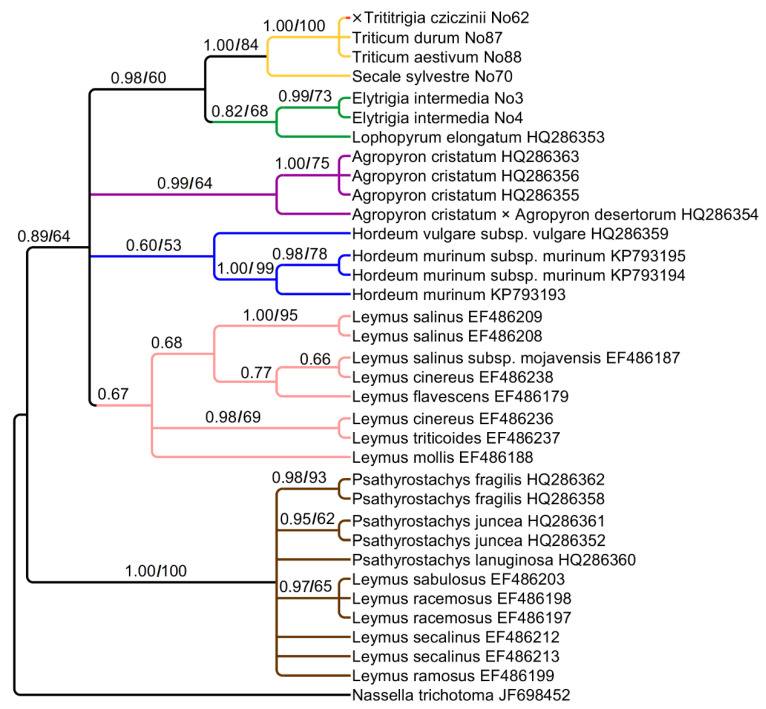
Phylogenetic tree showing the relationships of artificial hybrid ×*Trititrigia cziczinii*, its parental taxa, and allied genera according to the *trn*K–*rps*16 data (Sanger method). The first index is the posterior probability in Bayesian inference, the second is bootstrap value calculated by Maximum Likelihood analysis. When only one index is shown on the tree, it is the posterior probability index.

For independent assessment of phylogeny, we analyzed two chloroplast regions: *trn*K–*rps*16 and *ndh*F. Chloroplast sequences *trn*K–*rps*16 have 711 aligned positions. They form weakly to strongly supported clades (Figure 3). ×*Trititrigia cziczinii* has maternal genome of *Triticum aestivum* (PP = 1, BS = 100) falling into a clade with *Secale sylvestre* Host (PP = 1, BS = 84). The clade containing ×*Trititrigia + Triticum + Secale* L. is sister to the clade of *Elytrigia intermedia* + *Lophopyrum elongatum* (Host) Á. Löve (PP = 0.98, BS = 60).

There are the clades that comprise *Agropyron* Gaertn. (PP = 0.99, BS = 64), *Hordeum* (PP = 60, BS = 53), and *Leymus* (PP = 0.67) that form large clade along with *Triticum* and *Elytrigia intermedia* (PP = 0.89, BS = 64). The genus *Psathyrostachys* forms the second large clade (PP = 1, BS = 100). This clade also includes some species of the genus *Leymus* from the complexes *L. racemosus* (Lam.) Tzvelev and *L. sabulosus* (M.Bieb.) Tzvelev.

**Figure 4:**
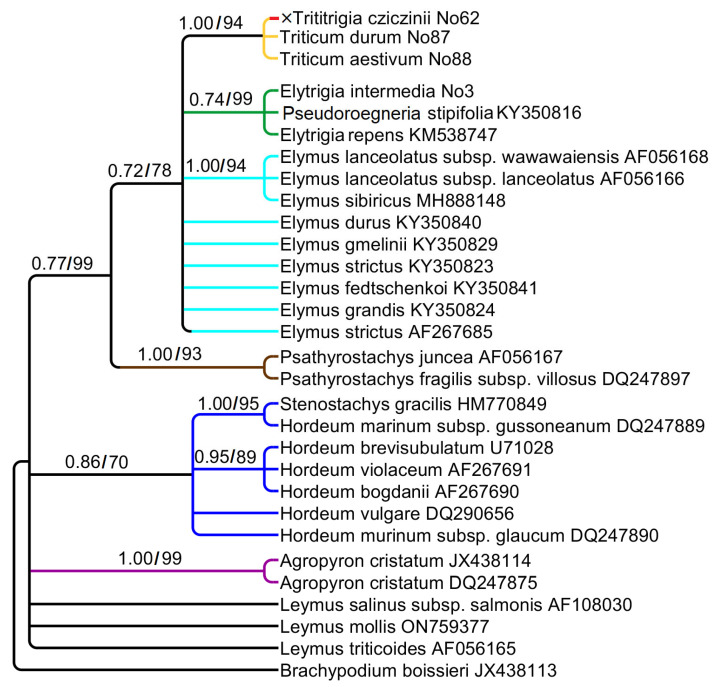
Phylogenetic tree showing the relationships of artificial hybrid ×*Trititrigia cziczinii* parental taxa, and allied genera according to the *ndh*F data (Sanger method). The first index is the posterior probability in Bayesian inference, the second is bootstrap calculated by Maximum Likelihood analysis.

The studied sequences of *ndh*F comprise 663 aligned positions. The phylogenetic tree based on *ndh*F sequence data is rather similar to the *trn*K–*rps*16 tree but less supported (Figure 4). ×*Trititrigia cziczinii* has the chloroplast sequences of *Triticum aestivum* and *Triticum durum* (PP = 1, BS = 94). *Elytrigia intermedia* forms a clade with *Pseudoroegneria stipifolia* and *Elytrigia repens* (PP = 0.74, BS = 99). Species of the genus *Elymus* occupy an uncertain position in the large clade *Triticum + Elytrigia + Elymus*. The genus *Psathyrostachys* has a sister position to this clade (PP = 0.77, BS = 99).

*Stenostachys gracilis* (Hook.f.) Connor falls into a clade with the species of *Hordeum* (PP = 0.86, BS = 70) and is monophyletic with *Hordeum marinum* Huds. subsp. *gussoneanum* (Parl.) Thell. (PP = 1, BS = 95) possibly revealing H-genome. Some *ndh*F sequences of the genus *Leymus* have an uncertain position on the tree.

### 2.2. NGS Data, Ribotype Network and Tree

For more precise tracking hybridization events, we built a network of the ITS 1 ribotypes obtained by NGS analysis. The alignment of 257 sequences consists of 346 positions. Major ribotypes (more than 1% per rDNA pool) are presented in Table 2.

**Figure 5:**
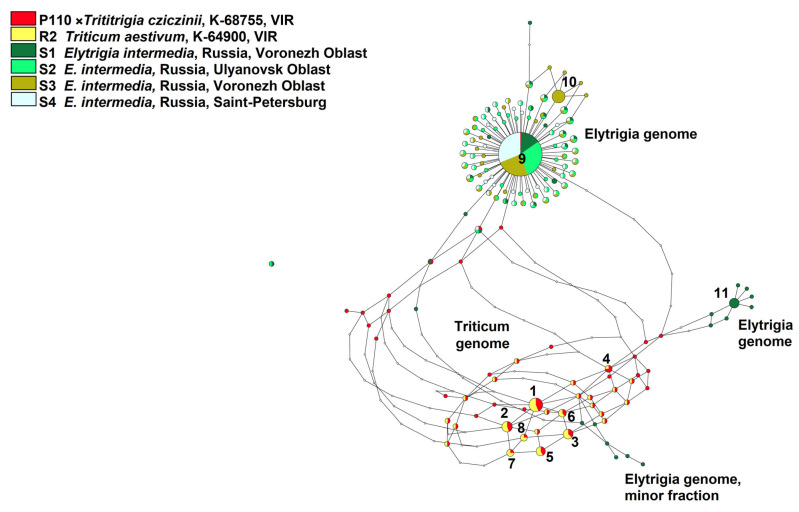
Ribotype network of the artificial hybrid ×*Trititrigia cziczinii* and its parental taxa based on NGS data built by Statistical Parsimony algorithm. The diameter of the circles is proportional to the percentage of the major ribotype to the total number of reads in the sample.

Ribotype network made by statistical parsimony method shows that the major ribotypes of ×*Trititrigia cziczinii* belongs to *Triticum aestivum* (Figure 5) and are identical to its major ribotypes. In this case, we considered ribotypes with more than 1% of reads per the rDNA pool of the sample as major ones. The most part of the studied rDNA from ×*T. cziczinii* is related to *T. aestivum*.

Only a minor fraction of ×*T. cziczinii* ribotypes (64 reads) is identical to the main ribotype of all studied *Elytrigia intermedia* samples (1990 reads, 25% of the whole rDNA pool; 4225 reads, 49%; 3792 reads, 41%, and 4392 reads, 53%, respectively). The second major ribotype of *E. intermedia* from Voronezh Oblast, sample S1 (600 reads, 8%) is rather distantly related to other ribotypes of the studied *E. intermedia*. The ribotypes of *E. intermedia* sample S1 also form a small subnetwork that is more closely related to *Triticum*-ribotypes than the main ribotype of *E. intermedia*. Additionally, *E. intermedia* sample S3 from Voronezh Oblast has a specific second major ribotype (1326 reads, 14%) that is closely related to the main ribotypes of all studied *E. intermedia*.

**Figure 6:**
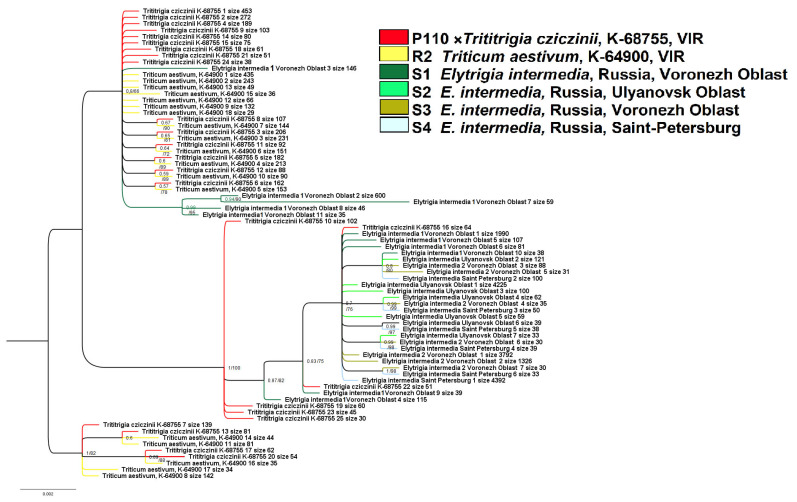
Phylogenetic tree of the studied ITS 1 ribotypes depicting origin of the artificial hybrid ×*Trititrigia cziczinii*. The first index on the tree node is a posterior probability (Bayesian inference); the second is a bootstrap value (ML method).

The phylogenetic tree of the studied ribotypes splits into three large clades (Figure 6). In this case, we set a threshold of 30 reads per rDNA pool. The first clade unites major ribotypes of ×*T. cziczinii* with these of *Triticum aestivum* (PP = 0.8, BS = 66).

Ribotypes of *Elytrigia intermedia* S1 (Voronezh Oblast) occupy an uncertain position within this clade. A separate subgroup within this large clade is formed by ribotypes of *E. intermedia* S1 (PP = 0.99, BS = 95) including the second major ribotype (600 reads). The next large clade (PP = 1, BS = 100) on the ribotype tree comprises the most part of the ribotypes of *E. intermedia* and some ribotypes of ×*Trititrigia cziczinii.* Only two ribotypes of ×*T. cziczinii* fall into subclade 7) with *E. intermedia* ribotypes, other ribotypes of ×*T. cziczinii* form a polytomy. The third small clade consists of the ribotypes of ×*T. cziczinii* and *Triticum aestivum* (PP = 1, BS = 82).

### 2.3. NGS Data, Ribotype Composition of the Studied Samples

**Figure 7:**
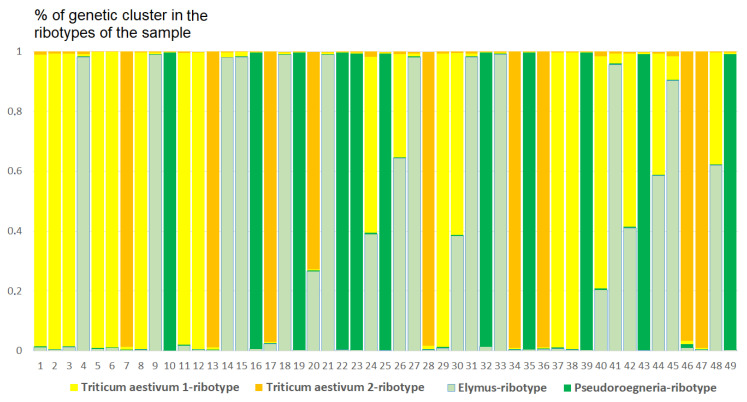
Genetic clustering of ×*Trititrigia cziczinii* (K = 4). The genetic clusters correspond to the probable ancestral ribotypes within the studied sample. They are named after the most similar sequences from the Genbank database. The number of columns corresponds to the number of ribotypes within the sample.

Analysis of the ribotype sets in studied samples performed by Structure software clustering revealed different quantities of the possible ancestral ribotypes in ×*Trititrigia cziczinii* (Figure 7). An estimated number of genetic clusters reflects ribotype composition within a given sample, and not between samples.

The studied sample of ×*Trititrigia cziczinii* has four estimated ancestral ribotypes (K = 4) in its rDNA. Two estimated ancestral ribotypes belong to *T. aestivum*-group, and one belongs to *Pseudoroegneria* (Nevski) Á. Löve (=diploid *Elytrigia*); the most similar species to *Pseudoroegneria*-ribotype is *P. stipifolia*. The fourth ancestral ribotype is *Elymus*-like, the most similar to ×*Elyhordeum kirbyi* M.P.Wilcox and *Elymus caninus* L.

**Figure 8:**
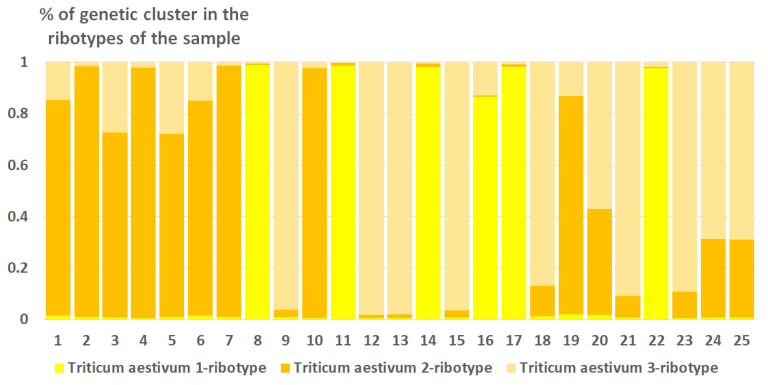
Genetic clustering of *Triticum aestivum* (K = 3). The genetic clusters correspond to the probable ancestral ribotypes within the studied sample. They are named after the most similar sequences from the Genbank database. The number of columns corresponds to the number of ribotypes within the sample.

Ribosomal DNA of *Triticum aestivum* consists of three estimated ancestral ribotypes (K = 3); all of them belong to the *T. aestivum*-ribotype family (Figure 8).

**Figure 9:**
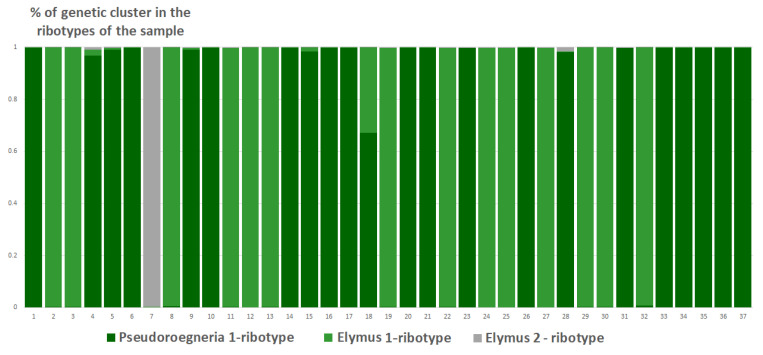
Genetic clustering of *Elytrigia intermedia*, from Voronezh Oblast, Russia, sample S1 (K = 3). The genetic clusters correspond to the probable ancestral ribotypes within the studied sample. They are named after the most similar sequences from the Genbank database. The number of columns corresponds to the number of ribotypes within the sample.

**Figure 10:**
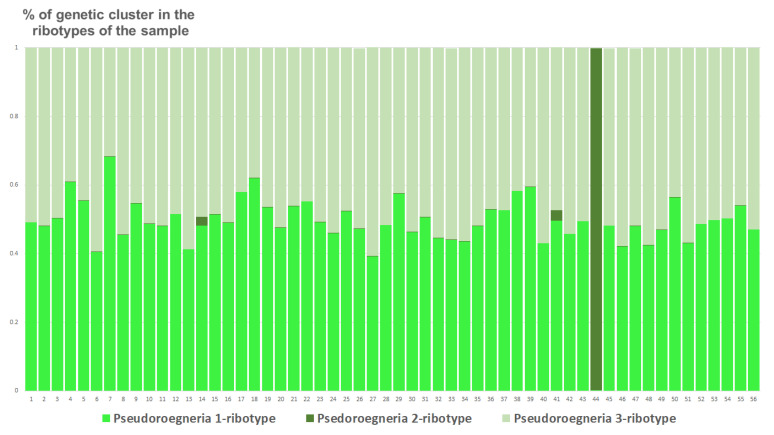
Genetic clustering of *Elytrigia intermedia*, sample S2 from Ulyanovsk Oblast, Russia (K = 3). The genetic clusters correspond to the probable ancestral ribotypes within the studied sample. They are named after the most similar sequences from the Genbank database. The number of columns corresponds to the number of ribotypes within the sample.

**Figure 11:**
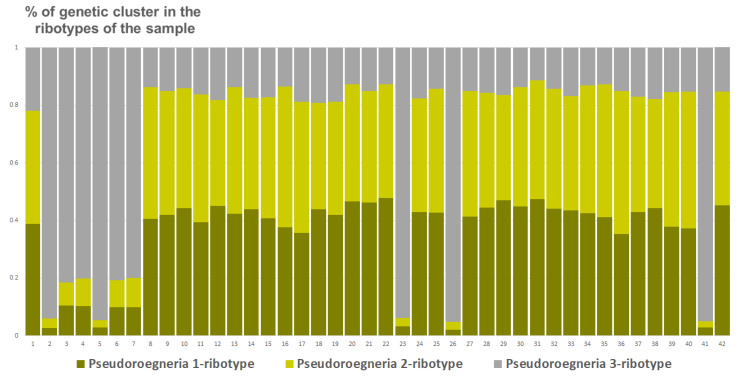
Genetic clustering of *Elytrigia intermedia*, from Voronezh Oblast, Russia, sample S3 (K = 3). The genetic clusters correspond to the probable ancestral ribotypes within the studied sample. They are named after the most similar sequences from the Genbank database. The number of columns corresponds to the number of ribotypes within the sample.

Studied *Elytrigia intermedia* samples differ in the number of estimated ancestral ribotypes. The first estimated ancestral ribotype of the first sample of *E. intermedia* from Voronezh Oblast (S1) belongs to *Pseudoroegneria*-ribotype group (K = 3); the closest species is diploid *Pseudoroegneria stipifolia* (Figure 9). The second estimated ancestral ribotype of *E. intermedia* S1 is similar to those of *Elymus nevskii* Tzvelev and ×*Elyhordeum kirbyi* (Figure 9). The third probable ancestral ribotype is similar to ×*Elyhordeum kirbyi* and *Elymus caninus* ITS sequences (Figure 9). Two studied samples of *Elytrigia intermedia*, S2 and S3 from Ulyanovsk and Voronezh Oblast, Russia, respectively, have three estimated ancestral ribotypes in their rDNA (K = 3 for both samples, Figure 9, Figure 10 and Figure 11). All these ribotypes belong to the *Pseudoroegneria*-ribotype family.

**Figure 12:**
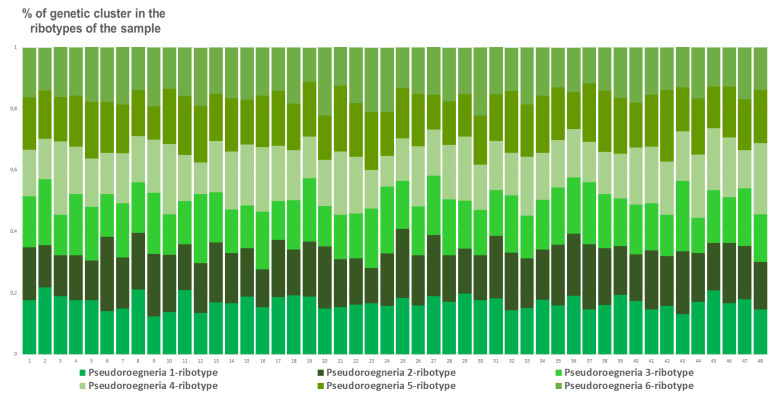
Genetic clustering of *Elytrigia intermedia*, from St. Petersburg, Russia, sample S4 (K = 6). The genetic clusters correspond to the probable ancestral ribotypes within the studied sample. They are named after the most similar sequences from the Genbank database. The number of columns corresponds to the number of ribotypes within the sample.

The sample of *E. intermedia* from St. Petersburg, Russia, has the most structure of the estimated ancestral ribotypes. It contains six estimated ancestral ribotypes (K = 6). They all are similar to *Pseudoroegneria* (mostly *P. stipifolia*) (Figure 12).

## 3. Discussion

According to modern data, distant hybridization, accompanying polyploidization, is one of the most important directions of plant evolution. Recent molecular phylogenetic studies indicated that all contemporary flowering plants have undergone several acts of polyploidization in their evolutionary history [26,27]. The grass family, for example, passed through at least three rounds of polyploidization affecting main clades of the grasses [28]. Furthermore, intergeneric (and probably even intertribal) hybridization between the species occurred on the border of the tribes of Pooideae subfamily—former tribes Poeae and Aveneae [29,30,31,32,33]. The genera *Deschampsia* P.Beauv., *Vahlodea* Fr., *Aira* L., and *Avenula* (Dumort.) Dumort. (members of subtribe Airinae Fr.) could originate from intercrossing between relatives of *Avena* L. (Aveneae chloroplast group—[30,33]) and species from the tribe Poeae s. str. related to *Festuca* L. [29,32]. The genus *Psathyrostachys*, a member of the important tribe Hordeeae, probably hybridized (or accessed genes via horizontal transfer) with *Bromus* L. from allied tribe Bromeae Dumort. [16,20]. Thus, the grass species are very complex systems containing different genomes received from distantly related taxa, and these genomes can be in a state of balance. Our study can shed more light on the events of distant hybridization whereas morphological features can be hidden due to prolonged introgression.

The history of studying the genomic structure of the wheat tribe goes back decades. According to cytogenetic data, the species of *Triticum* (wheat) consist mainly of three subgenomes—A, B, and D [34,35,36]. D-genome was probably inherited from *Aegilops tauschii* Coss. [25,37,38,39,40,41]. Diploid wheat *T. urartu* Thumanjan ex Gandiljan was a donor of A-genome whereas some unidentified *Aegilops* species gave origin to B-genome [25]. *Triticum aestivum*, parental species for ×*Trititrigia cziczinii*, is hexaploid with the genome structure ABD [20,34,35,42,43]. The genus *Elytrigia* is taxonomically complicated; some species were transferred from this genus to other genera [17,18,43,44]. It was divided into several genera according to its genomic structure: *Pseudoroegneria* (St), *Lophopyrum* Á. Löve (E), *Thinopyrum* Á. Löve (J), *Elytrigia* (EJSt), and *Elymus* (StH) [17]. Dewey [18] treated *Elytrigia* s. l. as three independent genera: *Pseudoroegneria* (St), *Thinopyrum* (E or J), and *Elytrigia* (StX). Some studies identified similarity between E and J-genomes [45,46,47,48]. In addition, some researchers treated *Elytrigia repens*, a type species of the genus as *Elymus repens* since it has StStStStHH genomes [43,44,49,50]. As a result, the position of the genus *Elytrigia* turns out to be more complex and confusing than in the old systems built on morphological criteria. However, in this case, it seems to us more correct to use the morphological criterion to determine the boundaries of the genus *Elytrigia.* At the same time, we acknowledge some taxonomic clarifications, particularly concerning diploids (*Pseudoroegneria*). As for morphology, the species of the genus *Elytrigia* s. l. have glumes keeled at the upper part; sessile spikelets, a dent at the base of the glume, and their inflorescences are erect. Diploid species that now belong to *Pseudoroegneria* differ by their caespitose form. *Elytrigia intermedia*, a parent for ×*Trititrigia cziczinii*, is a hexaploid species with ESt genome combination [24].

The hybrid ×*Trititrigia cziczinii* studied by us is artificial, meaning that we can clearly identify the parental taxa and the genomic contribution of each. According to NGS data (ITS1 sequences), it is the ribotypes of *Triticum aestivum* that are most represented in the rDNA of the hybrid (Figure 5 and Figure 6). Ribosomal DNA of *T. aestivum*, in turn, originated from *Triticum* s. str. (A-genome from *T. urartu*) based on our analysis and not from a hypothetical progenitor from the genus *Aegilops*. The dominance of *Triticum aestivum* rDNA in the hybrid is probably due to backcrosses with this species undertaken by breeders to stabilize the genome of the distant hybrid. Mechanisms of selection between rDNA from different genomes have not yet been fully studied. According to modern data, nucleolar dominance involves various mechanisms of intranuclear regulation, primarily selective methylation of rDNA loci [25]. Dysploidy in plants (i.e., chromosome elimination in complex distant hybrids) can occur in two different ways [51]. In the artificial hybrids of two evolutionary different large clades, as occurs in hybrids *Triticum* × *Zea* L., loss of all maize chromosomes takes place during the first few embryonic divisions [52]. In octoploid hybrids of rye and wheat (triticale), all rye chromosomes remain, and the loss of chromosomes of *Triticum* goes on for several generations [53]. In the case of ×*Trititrigia*, chromosomes of *Triticum* are predominant (42 in the stabilized cultivar, [6]) and thus can determine genomic constitution of the hybrid. The change in the length of the IGS in the rDNA of the hybrid genome also plays a role [54,55]. Previous studies established the dominance of the A-genome in artificial and natural wheat polyploids [25]. Our result also supports AFLP data that clearly indicated predominance of the wheat genome in the genotype of ×*Trititrigia cziczinii* [56].

On the contrary, only a minor fraction of rDNA of ×*Trititrigia cziczinii* is identical to the main ribotype of *Elytrigia intermedia,* the other parent (Figure 5 and Figure 6). According to NGS analysis, *E. intermedia* has St-genome as the main in its rDNA. Most probably, it was inherited from diploid species of *Pseudoroegneria*; for example, European *P. stipifolia*. We found other minor ribotypes that do not belong to the St-genome only in *E. intermedia*, sample S1 (Voronezh Oblast, Russia). This sample of *E. intermedia* was collected from the slopes of the bank of the Khoper River in contrast to the samples collected on chalk outcrops (S2) and a steppe slope (S3). They are closer to the ribotypes of *Triticum aestivum* than to those of *Pseudoroegneria* on the phylogenetic trees (Figure 5 and Figure 6) but NCBI search (https://www.ncbi.nlm.nih.gov/nuccore/?term= (accessed on 23 June 2025)) results give the greatest similarity to the sequence of tetraploid (2n = 28) *Elymus nevskii*. We can assume that the second major ribotype of *Elytrigia intermedia* is a St-genome variant from some polyploid species of *Elymus* and from diploid *Pseudoroegneria* since *Elytrigia/Elymus* clade is itself sister to the clade of *Triticum* s. l. + *Secale* in recent phylogenies [20]. We can interpret this finding as the trace of introgression in one of the populations of *E. intermedia*.

Chloroplast sequence data clearly shows that the hybrid ×*Trititrigia cziczinii* has the maternal genome of *Triticum aestivum* (Figure 3 and Figure 4). This may be due to the way this artificial hybrid was stabilized: it was obtained through repeated introgressive crosses with *Triticum aestivum* to secure beneficial traits [5,6,57]. Nevertheless, surprisingly, analysis of ETS sequences revealed the proximity of ×*T. cziczinii* to *Elytrigia intermedia* (Figure 2). This is probably caused by significant reorganization of rDNA in the genome of the artificial hybrid and, as a consequence, the formation of chimeric sequences in some regions of rDNA. Selective amplification of minor rDNA sequences due to primer specificity may also play a role. This interesting fact tells us that *Elytrigia*-related sequences can retain important functions in a complex genome of the hybrid. Maternal genome of *Elytrigia intermedia* may have different origins either from *Pseudoroegneria* or from *Lophopyrum* species [24,58]. Our data do not contradict these findings.

Speaking of the relationships between ×*Trititrigia cziczinii*, parental species and allied taxa, we can find many traces of hybridization in the tribe Hordeeae as well as the clades that more or less correspond to the main genera taken into our work. Species of the genus *Pseudoroegneria* are not very close to polyploid *Elytrigia* and *Elymus* on the ITS tree (Figure 1). Thus, rDNA of *Elytrigia intermedia* is probably changed compared with its putative donor, *Pseudoroegneria*. The group of Arctic and Siberian species of *Elymus* (*E. hyperarcticus* (Polun.) Tzvelev, *E. sajanensis* (Nevski) Tzvelev) forms a separate subclade on the tree (Figure 1) being a Eurasian subgroup 3 of Leo et al. [59]. This also points to the independent capture of St-genome in the genus *Elymus* and possible polyphyletic origin of *Elymus* and *Elytrigia* species*. Elytrigia repens* and *E. pseudocaesia* from Altai Mountains, Russia, form a distinct clade, and that most likely proves the separate origin of *Elytigia repens* and its affinity group. We also found a P-genome line that corresponds to the genus *Agropyron. Elytrigia lolioides* and *Elymus tauri*, surprisingly, also fell within this clade (Figure 1). *Elytrigia lolioides* is a tetraploid species with 2n = 28 ([60], for the Russian samples) or hexaploid, 2n = 42 [61]. P-genome sequences of *E. lolioides* were not found in the previous works [24]. We probably observe here more complicated and changing genome constitution in *E. lolioides* because other studies revealed an H-genome in this species instead of P [24]. Previous research considered *Elymus tauri* as a diploid with a St-genome specific to *Pseudoroegneria tauri* (Boiss. & Balansa) Á. Löve [24,62,63,64]. Nuclear rDNA shows the genus *Psathyrostachys* as the genome donor for *Leymus*, genome Ns (Figure 1). Chloroplast sequences trnK–rps16 show division of the genus into two groups based on the maternal genome (Figure 3). *Leymus secalinus* (Georgi) Tzvelev and related species—*L. racemosus* (Lam.) Tzvelev and *L. sabulosus* (M.Bieb.) Tzvelev share genome Ns. *Leymus salina* (M.E.Jones) Á. Löve with allied species—*L. cinereus* (Scribn. & Merr.) Á. Löve, *L. flavescens* (Scribn. & J.G.Sm.) Pilg., *L. mollis* (Trin.) Pilg., and *L. triticoides* (Buckley) Pilg. have a distant maternal genome, Xm [65]. It is interesting that rDNA of Russian samples of *Leymus arenarius* Hochst. can carry both Xm and Ns genomes [66].

## 4. Materials and Methods

### 4.1. Plant Materials

Samples of the artificial hybrid ×*Trititrigia cziczinii* were taken from collection of the Federal Research Center N. I. Vavilov All-Russian Institute of Plant Genetic Resources (VIR). Samples of the parental taxa we obtained from collection of the Komarov Botanical Institute of the RAS (LE). In addition, we analyzed some marker sequences of the genera of the Hordeeae tribe from the NCBI database (https://www.ncbi.nlm.nih.gov/nuccore/?term= (accessed on 23 June 2025)) These taxa are related to the studied species and can represent main lines of the tribe evolution. Studied samples are shown in Table 1.

### 4.2. DNA Extraction, Amplification, and Sanger Sequencing

Genomic DNA was extracted from dried leaf and seed material using a Qiagen Plant Mini Kit (Qiagen Inc., Hilden, Germany). Some marker regions were amplified and sequenced by the Sanger method. ITS1–5.8S rDNA–ITS2 region was amplified according to the following cycle: initial denaturation at 98 °C for 1 min, followed by 35 cycles of 98 °C for 15 s, 56 °C for 15 s, 72 °C for 30 s, and a final elongation of 72 °C for 5 min using ITS 1P [67] and ITS 4 [68] primers. Chloroplast fragment *trn*K–*rps*16 was amplified under the similar conditions using rps16–4547mod [69] and trnK5′r [70] primers but annealing temperature was 55 °C. Chloroplast gene *ndh*F was obtained with primers ndhF_F_mod and ndhF_R [[71], with modifications] according the same time parameters as the previous regions, and annealing temperature was 52–54 °C. Nuclear region 3′-ETS was amplified with primers ETS-2F and 18S_start_R_primer [14]. PCR protocol was with the same time parameters, and annealing temperature was 63 °C. The sequencing was performed at the Center for the collective use of scientific equipment “Cellular and molecular technologies for the study of plants and fungi” of the Komarov Botanical Institute, St. Petersburg, according to the standard protocol provided with a BigDyeTM Terminator Kit ver. 3.1 set of reagents on the ABI PRIZM 3100 sequencer (168 Third Avenue, Waltham, MA USA).

### 4.3. Molecular Phylogenetic Analysis of the Sequences Obtained by the Sanger Method

Chromatograms were analyzed with Chromas Lite version 2.01 (Technelysium Co. South Brisbane QLD 4101, Australia) and then the sequences were aligned with the aid of the Muscle algorithm [72] included in MEGA XI [73]. The best evolutionary models for each dataset were computed with the aid of jModeltest program v. 2.1.10 [74]. We took each of the data sets separately in Mr. Bayes 3.2.2 [75]: 1–1.5 million iterations, the first 25% of trees were excluded as “burn-in”. Evolutionary models were GTR + I + G for ITS and ETS data, TVM + G for *trn*K–*rps*16, and TVM + I for *ndh*F sequence data. Maximum likelihood analysis was performed by IQ-TREE 2.3.6 [76], with ultrafast bootstrap option, 1000 iterations and the same evolutionary models as in Bayesian inference.

### 4.4. Next-Generation Sequencing

For amplification of ITS1 rDNA, the following conditions were used: initial denaturation at 94 °C for 1 min, followed by 25 cycles of 94 °C for 30 s, 55 °C for 30 s, 72 °C for 30 s, and a final elongation of 72 °C for 5 min using ITS 1P [67] and ITS 2 [68] primers. PCR products were purified using AMPureXP (Beckman Coulter, Indianapolis, IN, USA). The libraries for sequencing were prepared according to the manufacturer’s MiSeq Reagent Kit Preparation Guide (Illumina) (https://www.illumina.com/products/by-type/sequencing-kits/cluster-gen-sequencing-reagents/miseq-reagent-kit-v3.html (accessed on 15 May 2025)). They were sequenced on an Illumina MiSeq instrument (Illumina, San Diego, CA, USA) using a MiSeq^®^ Reagent Kit v3 (600 cycles) with paired-end reading (2 × 300) following the manufacturer’s instructions. The fragments were amplified and sequenced at the Center for Shared Use “Genomic Technologies, Proteomics, and Cell Biology” of the All-Russian Research Institute of Agricultural Microbiology.

### 4.5. Molecular Phylogenetic Analysis of NGS Data

The obtained pool of raw sequences was trimmed by Trimmomatic [77] included in Unipro Ugene [78] as follows: PE reads; sliding window trimming with size 4 and quality threshold 12; and minimal read length 130. Then, paired sequences were combined and dereplicated and sorted by vsearch 2.7.1 [79]. The resulting sequences formed ribotypes in the whole pool of genomic rDNA; they were sorted according to their frequency. For our analysis, we established a threshold of 10 reads per pool of rDNA. The sequences were aligned using MEGA v. 11.0.13 [73]; a ribotype network was built in TCS 1.2.1 [80] by statistical parsimony method and then visualized in TCS BU [81]. In addition, we constructed a phylogenetic tree of the obtained ribotypes by Bayesian and Maximum Likelihood methods using GTR + G model. Bayesian analysis was conducted with 2–5 millions of generations by Mr. Bayes 3.2.2 [75]. ML analysis was conducted with the aid of IQ-TREE 2.3.6 [76]. In this case, we set a threshold of 30 reads per rDNA pool.

The artificial hybrid ×*Trititrigia cziczinii* as well as its parental species are polyploids. Thus, their rDNA fraction is inherited from different parental taxa and forms different families of ribotypes. The rDNA polymorphism depicts the probable origin of the ribotypes within a sample from ancestral taxa similar to that when geographic differentiation is revealed by analyzing gene sequences of different samples of a species. In this case, we can reveal the origin of ribotypes in the sample corresponding to different subgenomes. To analyze the ribotype pattern of each sample separately, we conducted a model-based clustering method using the program Structure 2.3 [82]. The sequence files in fasta-format were converted to the Structure input files by R script for diploid organisms https://sites.google.com/site/thebantalab/tutorials#h.e9y185vac91q (accessed on 23 June 2025). We tested the rDNA pool of each sample obtained via NGS for revealing single-nucleotide polymorphisms (SNPs) that can be phylogenetically significant. The genetic clusters computed by Structure more or less correspond to the ribotypes of the sample and reflect probable ancestral taxa that gave origin to the ribotypes of the studied species. Each run of Structure 2.3 [82] was carried out as follows: burn-in period of 10,000 replicates, 50,000 MCMC replicates after burn-in, 3 iterations of each burn-in computing, and K (number of hypothetic ancestral population) was set from 2 to 8. Then, we calculated the correct number of K with the aid of Evanno test [83] implemented in StructureHarvester Python Script for Python 3.13 [84]. The resulting K is a number of hypothetical ancestral ribotypes that are present in ×*Trititrigia cziczinii*; mostly, they represent the major ribotypes with their derivatives, but also some minor ribotypes. Results of the clustering were visualized in MS Excel 2016. We analyzed the ribotype pool of each sample separately; ribotypes within the samples are shown on the Figure 8, Figure 9, Figure 10, Figure 11 and Figure 12.

## 5. Conclusions

Our phylogenetic study performed with different DNA markers clarified the genome structure of the artificial intergeneric hybrid ×*Trititrigia cziczinii.* The predominant genome in the hybrid genome set is that of *Triticum aestivum*. This is consistent with the data obtained using the AFLP method. Such a pattern most likely originated due to backcrosses between early progenies of the artificial hybrid ×*Trititrigia cziczinii* and the parent species *Triticum aestivum*. Furthermore, here we see a remarkable example of rDNA elimination in one of the genomes during highly distant hybridization acts. Our data also demonstrate geographic heterogeneity in the genomic composition of one of the parental taxa, *Elytrigia intermedia*.

## Figures and Tables

**Table 1:** List of the Hordeeae species used in the study.

Species	Genomic combination (Haploid) [18,24,25]	Country of Origin and Location	Genbank Number, ITS	Genbank Number, NGS Data	Genbank Number, ETS	Genbank Number, *trn*K–*rps*16	Genbank Number, *ndh*F
*×Trititrigia cziczinii*	ABSt	Russia, Moscow Oblast	PX586197	PX586228–PX586275	PX562838	PX562828	PX562834
*Triticum aestivum*	ABD	Russia, Leningrad Oblast	PX586198	PX586459	PX562840	PX562831	PX562836
*Triticum durum*	AB	Russia, Samara Oblast	PX586199		PX562839	PX562830	PX562835
*Elytrigia intermedia* No 1	JrJvsSt	Russia, Voronezh Oblast	PX586203	PX586276–PX586312	PX562841		
*Elytrigia intermedia* No 2	JrJvsSt	Russia, Ulyanovsk Oblast	PX586202	PX586313–PX586368	PX562842		
*Elytrigia intermedia* No 3	JrJvsSt	Russia, Voronezh Oblast	PX586201	PX586369–PX586410	PX562843	PX562832	PX562837
*Elytrigia intermedia* No 4	JrJvsSt	Russia, St. Petersburg	PX586200	PX586411–PX586458	PX562844	PX562833	
*Agropyron desertorum*	P	Russia, Altai Republic	PX586211				
*Agropyron krylovianum*	?	Russia, Altai Republic	PX586209				
*Agropyron krylovianum*	?	Russia, Altai Republic	PX586210				
*Elytrigia geniculata*	StSt	Russia, Tyva Republic	PX586214				
*Elytrigia geniculata*	StSt	Russia, Altai Republic	PX586225				
*Elytrigia gmelinii*	St	Russia, Altai Republic	PX586222				
*Elytrigia gmelinii*	St	Russia, Altai Republic	PX586223				
*Elytrigia lolioides*	StH	Russia, Altai Republic	PX586212				
*Elytrigia pseudocaesia*	StStH	Russia, Altai Republic	PX586224				
*Elytrigia repens* var. *aristata*	StStH	Russia, Altai Republic	PX586220				
*Elytrigia repens* var. *aristata*	StStH	Russia, Altai Republic	PX586213				
*Elytrigia repens* var. *glauca*	StStH	Russia, Altai Republic	PX586221				
*Elytrigia repens*	StStH	Russia, Altai Republic	PX586217				
*Elytrigia repens*	StStH	Russia, Altai Republic	PX586218				
*Elymus hyperarcticus*	StH	Russia, Vrangel Island	PX586215				
*Elymus komarovii*	StH	Russia, Altai Republic	PX586219				
*Elymus vassiljevii*	StH	Russia, Taimyr Peninsula	PX586216				
*Hordeum brevisubulatum*	II	Russia, Altai Krai	PX586204				
*Hordeum brevisubulatum*	II	Russia, Altai Krai	PX586205				
*Hordeum brevisubulatum*	II	Russia, Altai Republic	PX586206				
*Hordeum roshevitzii*	I	Russia, Altai Republic	PX586207				
*Hordeum roshevitzii*	I	Russia, Altai Republic	PX586208				
*Leymus ramosus*	NsXm	Russia, Altai Republic	PX586227				
*Psathyrostachys juncea*	Ns	Russia, Altai Krai	PX586226				
*Secale sylvestre*	R	Azerbaijan				PX562829	

**Table 2:** List of the major ribotypes of hybrid ×*Trititrigia cziczinii* and parental species.

Species	Genome	Total Number of Reads	Ribotype Symbol (of the Major Ribotypes)	Number of Reads	% from the Total Number of the Reads
*×Trititrigia cziczinii*	ABSt?	6589	1	453	7
			2	272	4
			3	206	3
			4	189	3
			5	182	3
			6	162	2
*Triticum aestivum*	ABD	5002	1	435	9
			2	243	5
			3	231	5
			5	213	4
			6	153	3
			7	151	3
			8	144	3
*Elytrigia intermedia* No 1	ESt	7759	9	1990	26
			11	600	8
*Elytrigia intermedia* No 2	ESt	8496	9	4225	50
*Elytrigia intermedia* No 3	ESt	9263	9	3792	41
			10	1326	14
*Elytrigia intermedia* No 4	ESt	8215	9	4392	54

## Data Availability

Data are contained within the article.
